# Insight into resident burnout, mental wellness, and coping mechanisms early in the COVID-19 pandemic

**DOI:** 10.1371/journal.pone.0250104

**Published:** 2021-04-15

**Authors:** Dani Zoorob, Shivam Shah, Danielle La Saevig, Courtney Murphy, Shaza Aouthmany, Kris Brickman

**Affiliations:** 1 Department of Obstetrics and Gynecology, University of Toledo—College of Medicine and Life Sciences, Toledo, Ohio, United States of America; 2 University of Toledo—College of Medicine and Life Sciences, Toledo, Ohio, United States of America; 3 Department of Emergency Medicine, University of Toledo—College of Medicine and Life Sciences, Toledo, Ohio, United States of America; Iwate Medical University, JAPAN

## Abstract

**Background:**

Acute augmentation of stress and disruption of training, such as during the COVID-19 pandemic, may impact resident wellbeing.

**Objectives:**

We investigated how residents in various specialties in the United States were impacted by COVID-19 on mental wellbeing and resilience levels, and the methodology for coping with the stress incurred.

**Methods:**

In April 2020, the authors electronically surveyed 200 residency programs of all specialties nationally. The survey utilized two validated questionnaires to assess wellbeing and resilience, while investigating demographics and coping mechanisms. The authors used student t-test and ANOVA to quantitatively analyze the data.

**Results:**

The sample consisted of 1115 respondents (with an 18% response rate). Male gender & Age >39 years were associated with more favorable average well-being indices (both p<0.01). Regarding resources, institutional support (IS) appeared favorable for resident well-being (IS 2.74, SD1.96 vs NoIS 3.71, SD2.29, p<0.01) & resilience (IS 3.72, SD0.70 vs NoIS 3.53, SD0.73, p = 0.05). The effects of mindfulness practices (MP) were not statistically significant for improvement of wellness (MP 2.87, SD 1.99 vs No MP 2.76, SD 2.15, p = 0.85) or resilience (MP 3.71, SD 0.70 vs No MP 3.72, SD 0.68, p = 0.87).

**Conclusions:**

Findings highlight the critical importance of resident mental status in cases of augmented stress situations. Institutional support may contribute to promotion of resident wellbeing.

## Introduction

Resident physician mental wellness has increasingly been an area of study within the healthcare field. There is a large personal cost of burnout, fatigue, and declining mental health, in addition to the potential impacts of poor mental wellness on clinical decision making and patient outcomes [1–3]. Resident burnout is higher than burnout seen amongst similarly aged medical students, physicians, or college graduates and the rate of physician burnout is nearly 50% [4, 5]. Therefore, addressing mental wellness concerns during residency training is crucial. While there are many known contributing factors to resident mental wellness including sleep deprivation, lack of autonomy, environmental and workplace stressors, the current pandemic adds a novel burden.

COVID-19, the disease caused by SARS-CoV-2, was declared a global pandemic by the World Health Organization on March 11, 2020 [6]. In the month of April alone, during which the survey was administered, the Centers for Disease Control and Prevention noted an increase from 186,101 to 1,031,659 cases of COVID-19 in the United States. With the spread of COVID-19, many healthcare workers are faced with extreme working conditions and particularly challenging healthcare decisions. By providing care to patients, many residents are risking their personal safety. Concurrently, the stress of the pandemic, the rapidly rising burden of COVID-19 cases, and working in hazardous conditions can take a significant mental toll. In response to these new realities, programs and residents are forced to adapt. Beyond program endeavors to alleviate stress placed on residents, individual coping strategies and mindfulness techniques have become more important for mental wellness preservation. Mindfulness, “attending to relevant aspects of experience in a nonjudgmental manner”, is associated with improved clinician well-being, less burnout, and providing compassionate care [7, 8].

As the difficulty residents face in the fight against COVID-19 will continue to have significant implications for trainees and health care delivery, the objective of this study was to assess the resident response to the pandemic utilizing standardized instruments including the Brief Resilience Scale and the Mayo Clinic Resident Well-Being Index, in addition to specific questions regarding residency program support and mindfulness strategies. The reported survey results serve to capture the present impact of COVID-19 on residents and may be used to better align program support for stressful situations or environmental stressors.

## Methods

The study was approved by the ProMedica Institutional Review Board (IRB #20–043) with the data set collected and analyzed anonymously once completed. This study was based on a voluntary and anonymous, 25-question survey conducted between April 1–30, 2020. The survey was sent via email to 200 ACGME residency programs nationwide. The selection was based on listservs available to the IRB site. The survey utilized two validated questionnaires in conjunction with questions relating to demographics, mindfulness, and stressors. The survey queried residents on mindfulness practices used before and during the COVID-19 pandemic, major sources of additional stress due to the pandemic, and services offered by their institutions to reduce stress. The definition of mindfulness used was “maintaining a moment-by-moment awareness of our thoughts, feelings, bodily sensations, and the surrounding environment”. The six options for mindfulness routines queried included app-based mindfulness techniques, yoga or exercise, spending time with family, spending time with pets, arts such as music or painting, and psychological support by mentors. Free texting was allowed for additional methods used.

Two validated questionnaires were used, one specific to the residents and another applicable to the population as a whole. The Resident/Fellow Well-Being Index (RSWBI) is a validated 7-question tool that asks about feelings of burnout, symptoms of depression, and signs of fatigue to determine the level of wellness of each resident and/or fellow [9]. The scores range from 0–7, with higher scores having poorer wellness. A score of 2.64 is the average, based on a national survey, with a standard error of 0.02. RSWBI scores of 5 or more are considered “at risk” as they have a higher incidence of burnout, medical errors, and suicidal ideation. The Brief Resilience Scale (BRS) is a validated 6-question tool that measures a person’s resilience, which is the ability to recover from stress [10]. The score can range from 1–5, with 3.70 being the average with a standard deviation of 0.68. It has good internal consistency and test-retest reliability. Resilience scores less than 3.00 suggest low resilience while those above 4.30 suggest high resilience. The data was analyzed using SPSS (IBM SPSS Statistics for Mac, Version 27.0. Armonk, NY: IBM Corp). Quantitative analysis included use of student t-test and ANOVA, with statistical significance set at p < 0.05.

## Results

The total response rate was 18% (1,115 completed surveys out of 6181 total surveys sent to various specialty residency programs in the United States). Participants consisted of 655 (58.8%) females and 460 (41.2%) males. The most common age group was 25–29 years old, representing 452 (40.5%) of responses (Table 1). The highest number of respondents specialized in Emergency Medicine, Obstetrics & Gynecology, and Family Medicine. Many of the respondents (N = 457, 41.0%) reported greater than 20,000 confirmed COVID-19 cases in their respective state (Table 2).

**Table 1:** Population scores for wellness and resilience (SD = standard deviation).

	All Participants	Residents With Wellness Score ≥ 5	Residents With Resilience Score <3	Average Wellness Index (SD)	Average Resiliency Score (SD)
**Total**	N = 1115*	N = 270*	N = 137*	**2.84** (2.04)	**3.71** (0.69)
**Gender**		*p = 0*.*37*	*p = 0*.*24*	*p<0*.01**	*p<0*.01**
Men	**460** (41.2%)	**85** (31.5%)	**47** (34.3%)	**2.41** (2.05)	**3.85** (0.72)
Women	**655** (58.8%)	**185** (68.5%)	**90** (65.7%)	**3.12** (1.97)	**3.62** (0.66)
**Age**		*p<0*.*13*	*p = 0*.*89*	*p<0*.01**	*p = 0*.*89*
<25	**2** (0.2%)	**2** (0.7%)	**0** (0.0%)	**5.00** (0.00)	**3.67** (0.47)
25–29	**452** (40.5%)	**114** (42.2%)	**53** (38.7%)	**2.93** (1.99)	**3.70** (0.66)
30–34	**478** (42.9%)	**113** (41.9%)	**58** (42.3%)	**2.81** (2.05)	**3.72** (0.71)
35–39	**106** (9.5%)	**32** (11.9%)	**15** (10.9%)	**2.98** (2.16)	**3.69** (0.74)
>39	**77** (6.9%)	**9** (3.3%)	**11** (8.0%)	**2.22** (1.90)	**3.78** (0.76)
**Marital Status**		*p = 0*.*13*	*p = 0*.*94*	*p = 0*.*27*	*p = 0*.*48*
Single	**508** (45.1%)	**121** (44.8%)	**74** (54.0%)	**2.92** (1.98)	**3.66** (0.69)
Married/Cohabitate	**581** (51.6%)	**143** (53.0%)	**57** (41.6%)	**2.76** (2.09)	**3.76** (0.67)
Divorced	**26** (2.3%)	**6** (2.2%)	**3** (2.2%)	**3.00** (2.02)	**3.83** (0.70)
**Region**		*p = 0*.*09*	*p = 0*.*88*	*p<0*.01**	*p = 0*.*90*
Midwest	**324** (29.0%)	**71** (26.3%)	**45** (32.8%)	**2.70** (2.02)	**3.70** (0.69)
Northeast	**297** (26.6%)	**90** (33.3%)	**37** (27.0%)	**3.30** (2.04)	**3.69** (0.72)
South	**298** (26.7%)	**67** (24.8%)	**29** (21.2%)	**2.69** (2.00)	**3.72** (0.65)
West	**196** (17.6%)	**42** (15.6%)	**26** (19.0%)	**2.58** (2.01)	**3.75** (0.72)
**Mindfulness**		*p = 0*.*06*	*p = 0*.*30*	*p = 0*.*85*	*p = 0*.*87*
Practices	**793** (71.1%)	**193** (71.5%)	**99** (72.2%)	**2.87** (1.99)	**3.71** (0.70)
Does Not Practice	**322** (28.9%)	**77** (28.5%)	**38** (27.7%)	**2.76** (2.15)	**3.72** (0.68)
**Support Offered By Institution**		*p = 0*.*07*	*p = 0*.*23*	*p<0*.01**	*p = 0*.*05*
Yes	**997** (89.4%)	**217** (80.4%)	**114** (83.2%)	**2.74** (1.96)	**3.72** (0.70)
No	**118** (10.6%)	**53** (19.6%)	**23** (16.8%)	**3.71** (2.29)	**3.53** (0.73)

* Percentages are based on the column total used as denominator

**p< 0.05, significant

SD = standard deviation

**Table 2:** The distribution of survey participants relative to specialty correlated with wellness and resilience scores.

Specialty	All Participants (n, %)	Average Wellness Index (SD) *	Wellness Score ≥ 5* (n, %)	Average Resiliency Score (SD) *	Resilience Score <3* (n, %)
Anesthesiology	**73 (6.5%)**	**2.96** (2.27)	**21 (1.9%)**	**3.62** (0.74)	**12 (1.1%)**
Dermatology	**34 (3.0%)**	**2.21** (2.04)	**6 (0.5%)**	**3.70** (0.62)	**2 (0.2%)**
Emergency medicine	**206 (18.5%)**	**2.81** (1.94)	**52 (4.7%)**	**3.84** (0.68)	**21 (1.9%)**
Family Medicine	**163 (14.6%)**	**2.81** (1.98)	**40 (3.6%)**	**3.72** (0.70)	**21 (1.9%)**
Internal Medicine	**52 (4.7%)**	**3.71** (2.07)	**21 (1.9%)**	**3.37** (0.73)	**17 (1.5%)**
Neurology	**35 (3.1%)**	**2.57** (2.05)	**6 (0.5%)**	**3.63** (0.78)	**7 (0.6%)**
Obstetrics and Gynecology	**179 (16.1%)**	**3.13** (1.90)	**43 (3.9%)**	**3.69** (0.64)	**16 (1.4%)**
Other	**25 (2.2%)**	**2.56** (1.56)	**2 (0.2%)**	**3.75** (0.78)	**4 (0.4%)**
Pathology	**47 (4.2%)**	**2.51** (2.03)	**8 (1.7%)**	**3.41** (0.66)	**9 (1.8%)**
Pediatrics	**58 (5.2%)**	**2.78** (1.87)	**13 (1.2%)**	**3.71** (0.55)	**6 (0.5%)**
Physical Medicine and Rehabilitation	**30 (2.7%)**	**1.63** (1.97)	**3 (0.3%)**	**3.92** (0.74)	**3 (0.3%)**
Psychiatry	**47 (4.2%)**	**3.34** (1.91)	**13 (1.2%)**	**3.74** (0.58)	**4 (0.4%)**
Radiology	**73 (6.5%)**	**2.25** (1.98)	**10 (0.9%)**	**3.63** (0.77)	**10 (0.9%)**
Surgery or Surgical Subspecialty	**93 (8.3%)**	**3.03** (2.40)	**32 (2.9%)**	**3.88** (0.71)	**5 (0.4%)**
Total	**1115 (100%)**		**270 (24.2%)**		**127 (12.3%)**

*p<0.01, significant

**Fig 1:**
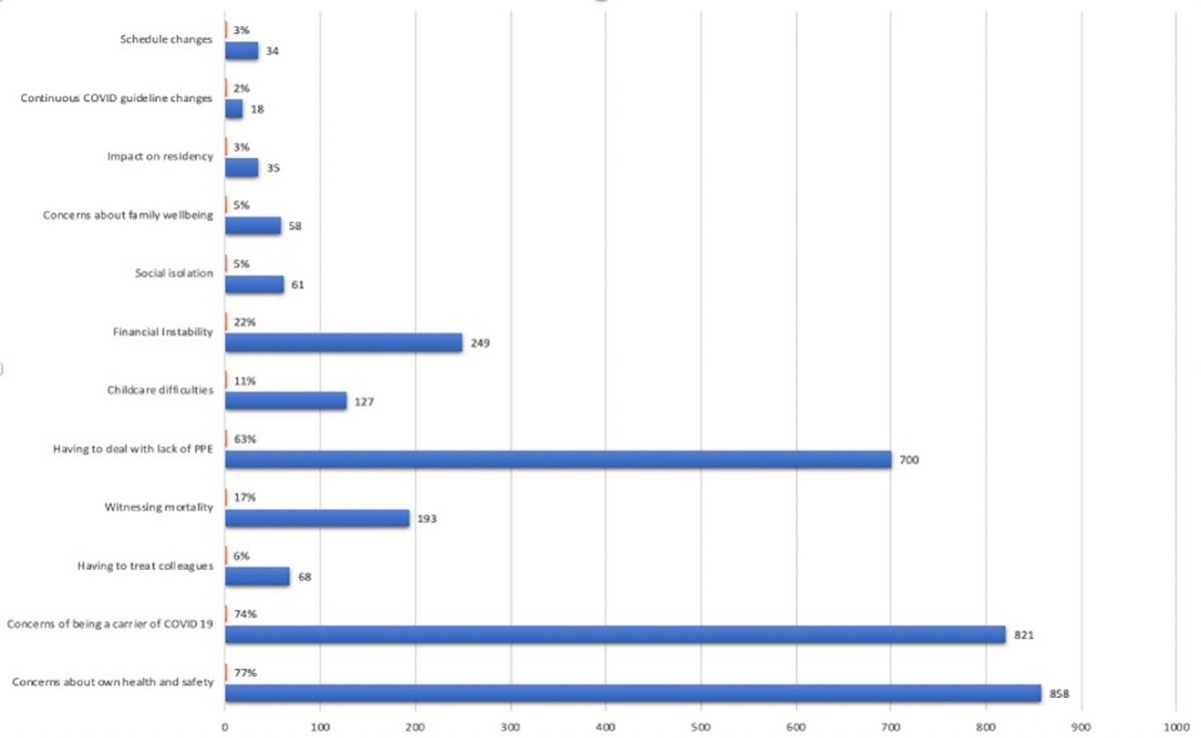
Major concerns of residents during the COVID-19 pandemic. (X-Axis represents the number of residents; Y-Axis represents various services offered; percentage is based on a 1115-person denominator).

Of all respondents, 793 (71.1%) reported using strategies to increase personal mindfulness, citing spending time with family and friends (n = 645, 58%) and exercising or yoga (n = 555, 50%) as the most common strategies (Table 1). Concerns about personal health and safety, being a potential COVID-19 carrier, and having lack of resources in the health care setting represented the 3 principal factors adding stress to respondents’ lives (77%, 74%, and 63%, respectively, Fig 1). Meal support (providing meals or discounts), residency program mentorship, and counseling services have been the most commonly offered support modalities that helped (27%, 27%, 8% respectively). Despite the services offered, 377 (34%) of respondents found none to be adequately helpful. Additionally, 13.6% reported falling asleep while inactive in a public place, 51.5% of respondents felt burned out, and 69.9% reported being distressed by emotional concerns regarding the pandemic.

## Discussion

The pandemic has impacted individuals worldwide, including healthcare workers. Residents, a subset of this group, were posed with additional unique challenges that related not just to the epidemic but to their training. In addition to the daily demands of residency programs outside of a global pandemic, resident physicians have had to work in environments where information was continuously evolving about how to best protect oneself or one’s patients, how to adapt and apply new guidelines, and some needing to redeploy to other services other than their specialty. With the institution of social distancing and quarantining, additional stressors emerged as unprecedented burdens amongst this group, particularly the sudden absence of social support, the limited access to childcare, and the risk of infecting family members in one’s home.

Evaluation of wellness and resilience scores while analyzing them based on specialty, indicates that internal medicine residents have the worst scores of all respondents (Table 2). Interestingly, internal medicine residents demonstrated the least resilience, while physical medicine and rehabilitation (PM&R) residents showed the most resilience. This deviation can most likely be attributed to the resident’s extent of patient involvement. Internal medicine residents come in frequent contact with COVID patients, while PM&R residents may have limited involvement.

While excluding confounders during the early pandemic season, resident wellness appears to have varied with COVID case rates in the various states. As the RSWBI tool assesses wellness within the previous month, overall better wellness scores correlated with states with lower COVID cases (<500), while increasing infection rates were more associated with concerning values (RSWBI≥ 5) (Table 3). States with higher than 20,000 cases had the overall highest participant percentage in the study as well as poorer wellness as an absolute number—within the past month (125 out of 270 with RSWBI≥ 5, 46%). The same states had the ‘lower / concerning’ resilience scores (score<3, 52 out of 137, 38%). These findings may be a result of selection bias, as residents who are most affected by COVID-related burnout may be most interested in responding to a survey that is relevant to their experiences. The concern stems from using incorrect metrics for evaluating the wellness of residents, especially when viewed simply in connection with anticipated future increases in infections, rather than addressing the issues that could impact wellness per se—such as resident exposure. Additionally, the validated tool used only assessed the status within the previous month and cannot identify confounders or factors that may impact wellness. This, however, may reliability of the scores reported.

**Table 3:** The distribution of survey participants relative to case count per state correlated with wellness and resilience scores.

Number of State Cases	All Participants (n, %)	Wellness Score ≥ 5* (n, %)	Resilience Score <3** (n,%)
<500	**27 (2.4%)**	**10 (0.9%)**	**1 (0.1%)**
500–1000	**53 (4.8%)**	**13 (1.2%)**	**8 (0.7%)**
1000–5000	**269 (24.1%)**	**53 (4.8%)**	**32 (2.9%)**
5000–10000	**171 (15.3%)**	**39 (3.5%)**	**25 (2.2%)**
10000–20000	**138 (12.4%)**	**30 (2.7%)**	**19 (1.7%)**
>20000	**457 (41.0%)**	**125 (11.2%)**	**52 (4.7%)**

*p<0.01, significant

**p = 0.73

This could help shed light on understanding the scores of the different specialties within the surveyed group. Currently, the only explanation for the decreased average resilience and increased average wellness indices is the relatively increased exposure for internal medicine residents to COVID patients. This has been substantiated by the similar score patterns seen in other specialties, such as anesthesiology, psychiatry, emergency medicine, family medicine and obstetrics and gynecology, that are more involved in positive or suspected COVID patient care.

Despite the pressures imposed by the pandemic, many institutions attempted to meet the needs of residents, such as through modifying coverage schedules and offering childcare assistance. Although statistically significant differences were noted on the wellness and resilience scales in those who were offered institutional support and those who weren’t, there was no difference between the groups who were “at risk” or had poorer resilience (Table 1). This suggests that whereas “healthier” residents may benefit from institutional support, the latter group may not be taking advantage of services offered and thus such support is possibly not be helping the ones who need it the most.

Having educational support and institutional resources has historically led to greater wellbeing and resiliency in learners [11]. The top services offered to residents that were reported to be most helpful during the early pandemic included meal support and discounts, program mentorship and personalized contact with leadership, and counseling services. Still, 42% of those offered services did not seem to use them or feel that the services were adequately helpful. However, there was no difference in RSWBI scores and the incidence of those “at risk” if the resident reported the support to be helpful or not (p = 0.06). This study’s findings are in accord with current literature as the methodology for affecting impact in residents is still in its infancy—despite the wellness efforts being amplified over the past few years.

Our study suggests a statistically significant correlation in variability in resilience and wellness based on gender (p<0.01 for both). However, this variation is not noted when specifically focusing on the “at risk” or reduced resilience segments (p = 0.37 and p = 0.24 respectively) (Table 1). It is critical to note that the findings are only a suggestion of variation among genders at one point in time–the previous month as per wellness tool validation. However, even before the pandemic, it has been reported that female residents demonstrated poorer wellness compared with male residents [9]. This is also found to be true outside of the medical workforce [12]. It is suggested that the poorer wellness scores in women seem are due to decreased self-compassion and focus on basic needs—which relies on feelings of autonomy, ability and competence, and the connection of professional relationships [13]. This finding is critical as it is necessary to identify methodology to assist with reduction of such a gender gap.

Age appears to correlate with wellness as well. Those older than 39 years old performed significantly better in the RSWBI than those who were younger (p<0.01); however, there was no significant difference in resiliency scores. Lin suggested that higher emotional intelligence is directly correlated with better resident well-being [14]. It has been shown that emotional intelligence increases with age, especially that one’s handling of stress matures upon reaching the mid-30s [15, 16]. This could explain our findings, although the aforementioned study was not specific to the US resident physician population. Marital status did not seem to have an impact on wellness or resiliency (Table 1).

The survey suggested no differences in resiliency nationwide among residents, although average RSWBI scores were significantly increased in residents in the Northeast region of the country (p<0.01), thus indicating poorer wellness (Table 1). There was also a slightly higher incidence of those “at risk” (a RSWBI ≥5) for residents practicing in the Northeast (p = 0.09, nonsignificant). This could be due to New York, New Jersey, and Massachusetts having had the highest number of cases per state by the end of the month of April [17]. Additionally, when compared with states that had 500–5000 cases, the wellness was poorer to those with >20,000 cases (p = 0.01). It is unclear though if the difference in wellness was present before or is specific to the pandemic as the location of living and locale of where medicine is practiced may impact wellness [18].

One of the biggest surprises of our study was that mindfulness played no significant role in the wellbeing of residents during the early stages of the pandemic (Table 1). Studies done prior to COVID-19 suggested that mindfulness improves well-being, particularly for those who are more stressed and more likely to become burned out [19–21]. It is unclear how the pandemic and social isolation measures have impacted the quality or type of mindfulness practiced by residents but a majority of residents cited spending quality time with loved ones as their common form of mindfulness while social isolation became a major stressor. Modifications in mindfulness routines and the assessment being early in the pandemic could have resulted in the non-significance reported by the residents surveyed.

It is essential to note that the validated questionnaires used were not tested specifically under circumstances where a pandemic was involved or for the purpose of analysis of resident performance under stress from a global disease. Thus, the applicability may not be optimal although no other assessment devices currently exist that are as specific. Irrespective, it is essential that conclusions alluded to in this manuscript are regarded as suggestions of patterns identified and not necessarily definitive.

There are some limitations to this research study that must be noted. First, we were unable to determine what the true number of residents receiving this survey and electing to respond to it was due to various reasons including the inability of some programs to forward it to their residents, inability to access the survey in time, deployment, and focus on patient care. Additionally, this was a voluntary study which opens the possibility of voluntary response bias. Although the survey was intentionally designed to require less than 5 minutes for completion in order to augment the likelihood of participation, it is possible that residents who were too busy may not have had ample time to complete this survey. To minimize selection bias, the survey was sent to a multitude of residency programs as well as to different specialties. The response rate was dependent on program directors/coordinators forwarding the survey link to their residents as well as residents accessing their emailed links. Additionally, the limitation of the mindfulness routines offered could have contributed to erroneous reporting, although free texting was permitted as needed to help limit this concern. However, significant strengths of the study lie in the large sample of surveys obtained as well as the use of the validated resilience and resident-specific wellness scores. Although the RSWBI tool has been validated for assessment only within the month prior to the evaluation and has not been tested for pandemic conditions per se, its use lies in that our study specifically addresses the early implications of the pandemic. Variations in wellness patterns may have become discordant as the disease spread and the pandemic persisted.

Future studies may need to assess the impact of specific routines of mindfulness on resident wellbeing as well as identify how institutions can better support resident wellness. Additionally, institutional support appears to have made a significant impact on wellness. It may be prudent to focus on this type of support when addressing wellness efforts for any environmental or problematic stressors.

## Conclusions

This study suggests that the implications of resident stressors in the early phase of the COVID-19 pandemic seemed to vary based on age, gender, and geographic location. Additionally, while mindfulness may not have contributed to improved mental wellbeing, institutional support was regarded as more valuable under such circumstances. Further studies can help shed light on variability between early and late pandemic resident wellness.

## Supporting information

S1 Data (XLSX)
